# Cochlear Synaptopathy: A Primary Factor Affecting Speech Recognition Performance in Presbycusis

**DOI:** 10.1155/2021/6667531

**Published:** 2021-08-06

**Authors:** Zhe Chen, Yanmei Zhang, Junbo Zhang, Rui Zhou, Zhen Zhong, Chaogang Wei, Jing Chen, Yuhe Liu

**Affiliations:** ^1^Department of Otorhinolaryngology Head and Neck Surgery, Peking University First Hospital, Beijing, China; ^2^School of Electronics Engineering and Computer Science, Peking University, Beijing, China

## Abstract

The results of recent animal studies have suggested that cochlear synaptopathy may be an important factor involved in presbycusis. Therefore, here, we aimed to examine whether cochlear synaptopathy frequently exists in patients with presbycusis and to describe the effect of cochlear synaptopathy on speech recognition in noise. Based on the medical history and an audiological examination, 94 elderly patients with bilateral, symmetrical, sensorineural hearing loss were diagnosed as presbycusis. An electrocochleogram, auditory brainstem responses, auditory cortical evoked potentials, and speech audiometry were recorded to access the function of the auditory pathway. First, 65 ears with hearing levels of 41-50 dB HL were grouped based on the summating potential/action potential (SP/AP) ratio, and the amplitudes of AP and SP were compared between the two resulting groups. Second, 188 ears were divided into two groups: the normal SP/AP and abnormal SP/AP groups. The speech recognition abilities in the two groups were compared. Finally, the relationship between abnormal electrocochleogram and poor speech recognition (signal-to-noise ratio loss ≥7 dB) was analyzed in 188 ears. The results of the present study showed: (1) a remarkable reduction in the action potential amplitude was observed in patients with abnormal SP/AP ratios; this suggests that cochlear synaptopathy was involved in presbycusis. (2) There was a large proportion of patients with poor speech recognition in the abnormal SP/AP group. Furthermore, a larger number of cases with abnormal SP/AP ratios were confirmed among patients with presbycusis and poor speech recognition. We concluded that cochlear synaptopathy is not uncommon among elderly individuals who have hearing ability deficits, and it may have a more pronounced effect on ears with declining auditory performance in noisy environments.

## 1. Introduction

Presbycusis, also known as age-related hearing loss, is the loss of hearing that gradually occurs in most individuals as they grow older. It can be defined as progressive, bilateral, symmetrical hearing loss ranging from high frequency to speech frequency that impairs an individual's communicative skills [1]. In China, hearing loss is the second most common nonfatal disease that significantly affects the quality of life [2]. At present, the number of elderly people (variably defined, but generally older than 60) in China has reached 160 million [3]. Approximately, 70% of the elderly who are over 60 years old and have a hearing impairment suffer from presbycusis [4]. A growing amount of epidemiological evidence indicates that presbycusis has a serious effect on the physical and mental health of the elderly, including on their cognitive ability, social communication ability, and independence in life [5]. The optimal evaluation and management of presbycusis have become a public health priority.

Experiencing greater levels of difficulty in speech recognition within adverse listening environments is the primary handicapping dysfunction among aging listeners. Previously, several researchers were focused on correlating declining auditory performance with the degeneration of cochlear hair cells. The development of therapeutic interventions was also concentrated on improving speech recognition through signal amplification. As a rule, in hearing aid fitting, when the average hearing thresholds exceeds 40 dB HL on an audiogram, amplification is indispensable. However, only one out of five people who could benefit from using a hearing aid actually wears one. The majority of adults aged between 55 and 74 years who were fitted for a hearing aid do not wear it since there have been complaints regarding the hearing aid not providing sufficient hearing-related benefits in complex listening situations [6]. At the time when information regarding the aging cochlear pathology was unclear, there was a widespread interest in attributing the worse auditory performance within adverse environments to a condition involving a lesion or deficit at a “more central” site, namely, central auditory processing disorder (CAPD) [7].

Nevertheless, recent animal studies have revealed that cochlear synaptopathy may be an early-onset contributor to auditory functional decline [8]. Cochlear synaptopathy affects the connection between inner hair cells (IHC) and results in low spontaneous discharge rate (SR) fibers, leading to a dysfunction in the low SR fibers, which in turn reduces the speech perception ability in a noisy environment [9]. Altschuler found that neither hair cell loss nor auditory brainstem response (ABR) threshold shifts were correlated with the loss of gap detection, loss of IHC-AN connections may account for some influences on decreased gap detection in the aging model [10]. A relationship between the loss of IHC-auditory nerve connections and decreased gap detection was observed in synaptopathy model mice [11]. In older CBA/CaJ mice ears, a steady decline in cochlear synaptic connections was observed from their youth (4 weeks) to their old age (144 weeks) and until death and was initially more intense in the apex but spread throughout the cochlear spiral with increasing age [8]. Evaluating the numbers of synaptic ribbons per IHC in the postmortem tissue of a human temporal bone revealed an age-related decrease from 13.3 synapses per IHC to as low as 2.0 synapses per IHC [12]. The results of recent experiments also suggest that the ABR wave-I amplitude, ABR wave-V latency, and summating potential (SP) to action potential (AP) ratio could be more sensitive measurements in underlying noise-induced cochlear synaptopathy [13–15].

It is undeniable that hair cell loss, synapsis loss, and neuron degeneration have a significant effect on presbycusis; however, the relative contribution of each of these factors is still unrevealed. Based on the results of animal and human temporal bone studies, we hypothesized that cochlear synaptopathy may greatly contribute to the higher difficulty levels that aging listeners experience in understanding speech in a noisy environment. Therefore, the present study was designed with the aim of identifying the role of cochlear synaptopathy in human presbycusis, which could provide valuable insights that could aid in developing comprehensive strategies to manage presbycusis.

## 2. Methods and Materials

### 2.1. Participants

A total of 94 patients who were between 60 and 90 years old were recruited from the Outpatient Department of Otorhinolaryngology Head and Neck Surgery of Peking University First Hospital. They were all native speakers (Mandarin) who were in good health with no histories of ear-related concerns, such as otitis media, sudden deafness, and vertigo; no histories of neurologic and cognitive disorders; and no long-term histories of noise exposure. In addition, all the subjects had a junior high school or higher education level and signed an informed consent form. To be eligible, all the participants underwent otoscopic examinations, and pure tone audiometry (PTA), acoustic immittance, ABR, and auditory cortical evoked potential (cAEP) measurements. The inclusion criteria were audiograms that showed bilateral, symmetrical, sensorineural hearing loss and an acoustic immittance of type A in both ears. Participants with remarkably abnormal ABR and cAEP were excluded. Our study and all the protocols used conformed to the *Declaration of Helsinki*, as reflected in an a priori approval by the Biomedical Research Committee of the Peking University First Hospital (2018-106).

### 2.2. PTA and Acoustic Immittance

Audiometric thresholds were measured using an Interacoustics audiometer. Pure-tone air-conduction thresholds were measured from 250 Hz to 8000 Hz; the hearing threshold was calculated as the average of 500 Hz, 1000 Hz, 2000 Hz, and 4000 Hz. Bone-conduction thresholds were acquired from 250 Hz to 4000 Hz. Tympanograms were obtained using the Maico Middle Ear Analyzer.

### 2.3. Speech Recognition in Noise (Quick-Sin)

The sentences that were used in this study to test the participants' hearing abilities were selected from the Mandarin Quick Speech-in-Noise (M-Quick SIN) Test materials [16]. The test sentences were broadcast by female announcers. The background noise used was 4-talker speech noise. Further, 78 sentences were compiled into 13 tables (11 test tables and 2 exercise tables), and each table included six sentences containing five keywords. The signal-to-noise ratio (SNR) was gradually reduced from the first sentence to the sixth sentence in each sentence table. The loss of SNR representing the number of decibels required to understand the speech information in a noisy background among the hearing loss patients was measured using the following formula:

(1) $$\begin{array}{c} \text{SNR loss}=24.5-R\;\left(\text{number of words recognized correctly}\right) \end{array}$$

The subjects were allowed to practice 2-3 sentences. The intensity of the stimulating sound was 30 dB above the hearing threshold of each subject, and the detailed process was outlined to the subject prior to testing.

### 2.4. Electrocochleography (EcochG)

Stimulus generation and the data acquisition of EcochGs were controlled by a GSI Audera AEP/CAEP computer system and software. The subjects' ear canals were prepped by scrubbing them with a 70% alcohol cotton swab. The forehead along the midline and ear lobe of the patients were completely degreased via scrubbing, after which the electrodes were attached. The electrode on the forehead was grounded, the same side of the reference electrode was placed on the ear lobe, and the recording electrode was placed on the surface of the eardrum. The impedance between the pairs of electrodes was *<*2 k*Ω*. The acoustic stimuli were delivered via silicone tubing connected to the earphones. The stimuli of 100 *μ*s-clicks were delivered at 97 dB nHL in an alternating polarity at the rate of 9.1 Hz. Electrical responses were amplified with a 10-3,000 Hz passband filter. Up to 3000 sweeps were averaged with artifact rejection enabled in the software program. The AP and SP peaks were defined through visual inspection by two experienced observers. We use “one point method” to extract AP and SP. SP is defined as the first negative peak and AP is the second negative peak. The graphic illustration is attached to the supplementary material (S1). The SP and AP amplitudes were defined as the difference between peak and baseline values (the lowest amplitude within the first ms).

### 2.5. Statistical Analysis

The SPSS 17.0 software program was used for the data analysis. An analysis of variance (ANOVA) and a correlation analysis were used to assess the effect of age on the hearing threshold and of the PTA on speech recognition in noise. A *T*-test and chi-square test were performed to compare the mean differences between the groups when there were two independent variables. *p* < 0.05 was considered statistically significant.

## 3. Results

### 3.1. Average Hearing Threshold Increases with Age

**Figure 1:**
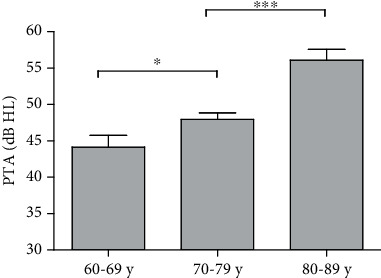
Comparison of PTA in different age groups. There were significant differences in PTA among the three groups. ^∗^*p* < 0.05. ^∗∗∗^*p* < 0.001. Abbreviations: PTA: pure tone average.

A total of 94 subjects (188 ears), including 49 female and 45 male subjects, aged between 60 and 90 years old (the youngest one was 60 years old while the oldest was 89 years old) were enrolled in this study. The results of the ANOVA revealed that the average hearing threshold increases with age and that there was a significant main effect of age on the hearing status (*F* = 16.83, *p* < 0.001, see S2). The difference between the groups was statistically significant (Figure 1).

### 3.2. Speech Recognition Ability Decreases with the Increase in Hearing Threshold

**Figure 2:**
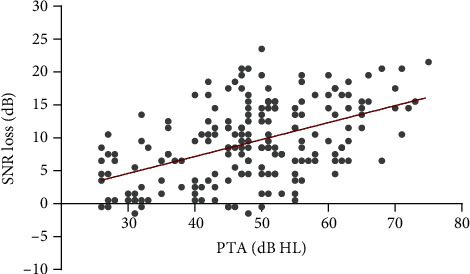
Scatter plot of the average hearing threshold and signal-to-noise ratio loss. Abbreviations: SNR: signal to noise ratio; PTA: pure tone average.

A scatter plot was constructed based on the average hearing threshold and SNR loss (Figure 2). When the average hearing threshold of the subject increases, the SNR loss gradually increases, implying that the speech recognition ability in noise declines. Even when the average hearing threshold was at the same level, the difference in the SNR loss was still very large.

### 3.3. A Lower AP Amplitude Results in Abnormal SP/AP Ratios

**Figure 3:**
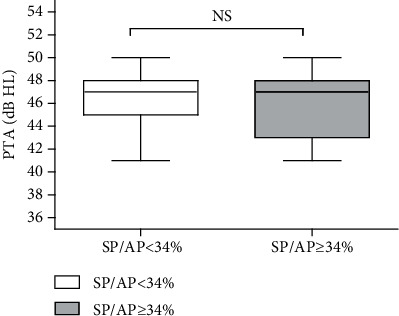
No observable differences in the hearing threshold was identified between the two groups. NS *p* > 0.05. Abbreviations: PTA: pure tone audiometry; SP: summating potential; AP: action potential; NS: nonsignificant.

The ratio between the waveform peaks generated by the hair cells (SP) vs. those generated by the cochlear neurons (AP), i.e., the SP/AP ratio, was identified as being normal when the ratio was <34%; otherwise, it was considered abnormal (according to our laboratory standard). We observed that 44% of the total of 188 ears exhibited an abnormal SP/AP. To investigate the cause of the high SP/AP ratios, 65 ears with hearing levels of 41-50 dB HL were selected for further analysis. They were divided into two groups based on the SP/AP ratio: the SP/AP <34% and SP/AP ≥34% groups. The basic characteristics of the 65 ears are presented in S3. No significant differences were found in the average hearing threshold between the two groups (*p* > 0.05, Figure 3).

**Figure 4:**
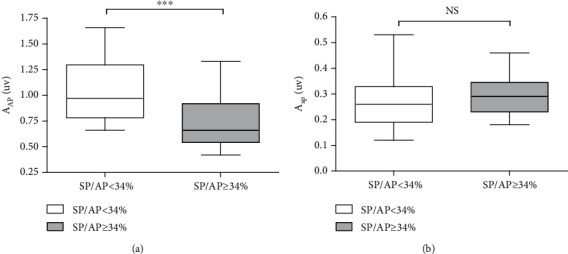
The amplitude of the AP and SP between the two groups. (a) A significant decrease in the AP amplitude of the patients with high SP/AP ratios. (b) There was no difference in the SP amplitude between the two groups. ^∗∗∗^*p* < 0.001. NS *p* > 0.05. Abbreviations: PTA: pure-tone audiometry; SP: summating potential; AP: action potential; NS: nonsignificant; A_AP_: amplitude of AP; A_SP_: amplitude of sp.

When the AP amplitude was compared between the two groups, a remarkable reduction in the AP amplitude was observed in the group with abnormal SP/AP ratios (*t* = 4.424, *p* < 0.001, Figure 4(a)). However, there were no significant differences in the SP amplitude between the two groups (*t* = 1.926, *p* = 0.064, Figure 4(b)).

### 3.4. Poor Speech Recognition in Noise Is Closely Related to a High SP/AP

**Figure 5:**
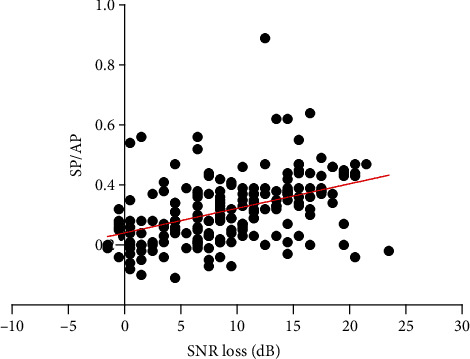
Scatter plot of SNR loss and SP/AP ratio. Abbreviations: SNR: signal to noise ratio; SP: summating potential; AP: action potential.

**Figure 6:**
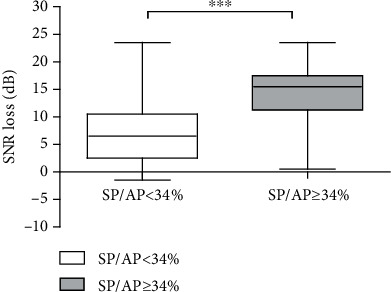
Comparison of the SNR loss in 188 ears. The SNR loss increased significantly in patients with high SP/AP ratios. ^∗∗∗^*p* < 0.001. Abbreviations: SNR: signal to noise ratio; SP: summating potential; AP: action potential.

Figure 5 presents the relationship between the SNR loss and SP/AP ratio with each point representing the data from a single ear. The scatter plot based on the data of a total of 188 ears indicates that people with poor speech recognition in noisy environments are more likely to have abnormal SP/AP ratios (Figure 5). The subjects with a high SP/AP exhibited a worse speech perception performance in a noisy background, which was verified in 188 ears (*t* = 22.15, *p* < 0.001, Figure 6). Moreover, the multivariable regression analysis shows SP/AP still have a significant correlation with SNR loss after the role of PTA has been factored in. SP/AP is an independent predictor of SNR loss and their correlation fits the following model:

(2) $$\begin{array}{c} \text{SNR loss}=3.156+9.612\text{ x }\left(\text{SP}/\text{AP}\right) \end{array}$$

**Figure 7:**
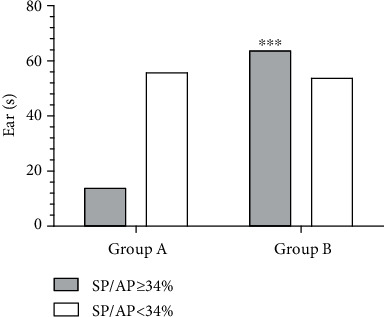
Comparison of the number of abnormal ears (SP/AP ≥34%) in the two groups. The results of EcochG in patients with poor speech recognition ability usually reveal a high SP/AP ratio. ^∗∗∗^*p* < 0.001. Abbreviations: EcochG: electrocochleogram; SP: summating potential; AP: action potential.

For further analysis, 188 ears were divided into two groups according to their speech recognition: Group A (SNR loss <7 dB) and Group B (SNR loss ≥7 dB). An SNR loss of ≥7 dB indicates poor speech recognition ability based on previous research [17]. A larger proportion of individuals had a high SP/AP ratio in Group B compared to in Group A (*χ*^2^ = 21.22, *p* < 0.001, Figure 7).

## 4. Discussion

Presbycusis is caused by the combination of multiple physiological factors, including the degeneration of the peripheral auditory organs, effects of noise, systemic diseases, and usage of ototoxic drugs [18]. Numerous complex factors that affect the hearing ability along with an overlap of pathologies exhibited by aging listeners pose a significant challenge in addressing the unique needs of the geriatric population with hearing impairments. Previous animal studies conducted on cochlear synaptopathy have opened the gate to a vast field in the area of research associated with presbycusis audiology. Thus, in this study, we set out to verify whether cochlear synaptopathy plays an important role in the auditory decline in human presbycusis. Our findings may help audiologists in prioritizing cochlear synaptopathy when it comes to making management decisions in treating aging listeners. Moreover, because cochlear implantation (CI) bypasses the sensory and synaptic partitions by directly stimulating the spiral ganglion cell, the health of the organ of Corti or synapse will not affect postoperative outcomes, and it is reasonable to speculate that the precise site of lesion causing presbycusis can help to determine at least a portion of outcomes of CI [19].

We recruited 94 presbycusis patients (188 ears) who met the study's inclusion criteria. In accordance with the results of previous studies, our study demonstrated that with age, a steady rise occurs in the hearing threshold. In addition, there is a certain negative correlation between the PTA and speech recognition ability in noisy environments.

### 4.1. Lower AP Amplitudes Imply That Cochlear Synaptopathy May Be an Important Component of Human Presbycusis

The first question that we sought to clarify in the present study was whether cochlear synaptopathy could potentially be a crucial clinical signature of presbycusis in human beings. The most commonly reported biomarker of synaptopathy is a marked reduction in the ABR wave-I amplitude at suprathreshold stimulus levels [20], while in humans, wave I, as measured via conventional ABR electrodes, is small and variable due to multiple factors. Hence, we used EcochGs to assess cochlear synaptic function. The first negative wave of AP was defined as N1 and is virtually the same component as wave I of the ABR and arises from the distal portion of the auditory nerve [21]. Previous study has confirmed that calculating the SP/AP ratio has evolved into an important approach for the diagnosis of Meniere's disease, an inner ear disorder that is characterized by fluctuating threshold shifts, episodes of vertigo, and tinnitus [22]. Based on this investigation, we found that 44% of the subjects presented with abnormal SP/AP ratios; nevertheless, these patients did not complain about any symptoms associated with Meniere's disease and were not diagnosed with endolymphatic hydrops. Studies have demonstrated that an enhanced SP/AP ratio in Meniere's disease is regulated by an SP elevation [23]. To further explore whether an AP reduction or SP elevation causes abnormal SP/AP ratios in these patients, we investigated the differences in the amplitudes of AP and SP. When hearing loss exceeds 60 dB HL, the cochlear potential recordings are questionable, and therefore, 65 ears with 41-50 dB HL were selected for further analysis in order to prevent the interference of the threshold level from affecting the EcochG. There are two distinct types of connections that connect hair cells in the peripheral axons of the spiral ganglion cells. The majority of the population (95%) exclusively targets the IHCs, and the others connect the outer hair cells (OHCs) [24]. Most of the OHCs are apoptotic when the hearing threshold exceeds 40 dB HL. Thus, in this study, we focused on the IHC area.

Figure 4 clearly indicates that a reduction in the AP contributes to the development of a high SP/AP ratio, which indicates the presence of quite a different mechanism from that of Meniere's disease. Otoacoustic emissions are mainly dominated by the OHCs that amplify the sound-evoked cochlear vibration [25], while the AP represents the summed activity of the auditory nerve fibers that connect the hair cells; therefore, recording EcochGs is a more reliable technique to evaluate the function of IHCs or of auditory nerve fibers in case of a hearing loss exceeding 40 dB HL. In this situation, a reduction in the AP amplitude reflects a nerve conduction block between the IHCs and auditory nerve terminal due to a presynaptic or synaptic dysfunction. The SP generated from the IHC receptor potentials could be used in distinguishing the presynaptic and postsynaptic dysfunctions [26]. Apparently, there is no difference in the SP amplitude. The results of these analyses demonstrate that deficits in the AP amplitude are caused by cochlear synaptopathy rather than a dysfunction in the mechanoelectric transduction of hair cells. Recent animal studies and human temporal bone histopathological findings have revealed that the cochlear synapses degenerate well before their cell bodies and that cochlear synaptopathy may be an early indicator of the degeneration of auditory organs associated with aging [8, 27]. Correspondingly, based on data acquired through EcochGs, our study clinically confirmed that cochlear synaptopathy might be an important component of human presbycusis.

### 4.2. Cochlear Synaptopathy May Be a Dominant Factor in Declining Auditory Performance in Noisy Environments among Patients with Presbycusis

As observed via the scatter plot representing the overall SNR loss and SP/AP ratio, speech recognition is associated with the SP/AP ratio in noisy environments. Some patients with high SP/AP ratios exhibited a poor auditory performance (Figure 6). The second question that we investigated in the present study was whether the poor speech recognition in a noisy environment is primarily caused by cochlear synaptopathy in patients with presbycusis. Consequently, we developed the following hypothesis: if the decline in speech recognition associated with presbycusis were mainly a result of cochlear synaptopathy, these patients would exhibit an abnormal SP/AP ratio. Therefore, in our study, the speech recognition and EcochGs of 188 ears with presbycusis were recorded and analyzed in order to confirm the above hypothesis.

The current study found that the majority of participants with an enhanced SP/AP ratio were present in the group exhibiting the worse auditory performance; further, the intergroup differences were highly significant. These results may be explained by the fact that cochlear synaptopathy may be a dominant factor involved in the declining auditory performance of elderly subjects.

There could be many factors that contribute to the declining auditory recognition in adverse listening situations in aging listeners. Indeed, many longitudinal and cross-sectional studies have demonstrated that the risk of CAPD in the elderly increases with age [28]. Age-related CAPD is defined as a peculiar deficit in the processing of auditory signals along with the central auditory nervous system [7]. The view outlining that the inability to perceive speech-in-noise is most likely to be related to CAPD in elderly individuals is pervasive [29]. Our study confirmed that cochlear synaptopathy is not uncommon among the elderly. The results obtained may be related to aging of the auditory pathways or superior centers or for other causes. We speculate that the development of cochlear synaptopathy might be a progressive process that is involved in the degeneration of the auditory pathway in the elderly and that it gradually develops from the cochlea and spreads to the auditory nerve.

The current classification of presbycusis based on audiometric tests and temporal bone pathology data was established by Schuknecht in 1993 [30]. However, these classic types of presbycusis are based on the morphology of the peripheral hearing organs rather than on the functional and central aspects of the auditory pathway. The classification of synaptopathy has not been described in detail. We hope to propose a new classification based on the synaptic subtype that has an important implication in clinical practice and hearing rehabilitation. In the future, the targeted screening of this subtype and, consequently, early intervention might result in lower debilitation levels resulting from presbycusis.

## 5. Conclusion

Our study recruited 94 presbycusis patients and observed a remarkable reduction in the AP amplitude in the patients with presbycusis with abnormal SP/AP ratios, which suggests the presence of cochlear synaptopathy in these presbycusis patients. Additionally, there was a large proportion of patients with poor speech recognition in the abnormal SP/AP group. We confirmed that cochlear synaptopathy could potentially be a dominant factor involved in declining auditory performance in noisy environments among patients with presbycusis.

## Data Availability

The data used to support the findings of this study were supplied by peking university first hospital under license and so cannot be made freely available. Requests for access to these data should be made to Dr. Chen (chenzhe_ent@163.com).

## Supplementary Materials

Supplementary Materials S1: “One point method” was used to extract AP and SP. SP is defined as the first negative peak and AP is the second negative peak. S2: the average hearing threshold increases with age and there is a significant difference on PTA in different groups. S3: there was no significant difference on PTA and age between the two groups.
