# Supplementary material for: Cochlear Synaptopathy: A Primary Factor Affecting Speech Recognition Performance in Presbycusis
Source: Biomed Res Int. 2021 Aug 6;2021:6667531. doi: 10.1155/2021/6667531 (PMC8367534; doi:10.1155/2021/6667531)
Supplement: Supplementary Materials — S1: “One point method” was used to extract AP and SP. SP is defined as the first negative peak and AP is the second negative peak. S2: the average hearing threshold increases with age and there is a significant difference on PTA in different groups. S3: there was no significant difference on PTA and age between the two groups. [file 6667531.f1.docx]

**[Supplementary](javascript:;) material**


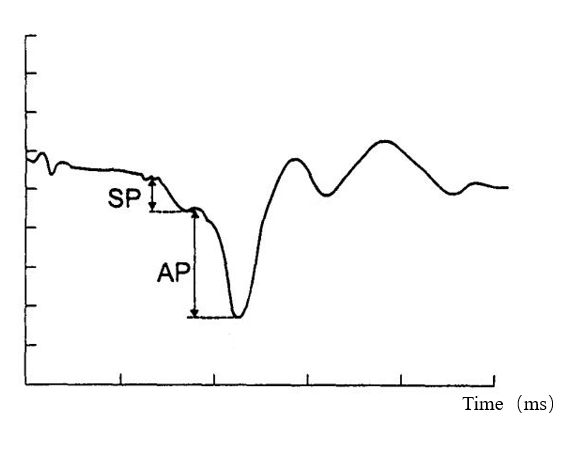


***S1. An example to illustrate how to define SP and AP.*** “One point method” was used to extract AP and SP. SP is defined as the first negative peak and AP is the second negative peak.

***S2. Comparison of PTA in different age groups (‾x ± s).*** The average hearing threshold increased with age and there was a significant difference on PTA in different groups.

| Age(year) | Ear(s) | PTA (dB nHL) |
| --- | --- | --- |
| 60-69 | 70 | 44.17±13.40 |
| 70-79 | 76 | 47.96±7.87 |
| 80-89 | 42 | 56.12±9.45 |
| Total | 188 | 48.37±11.44 |
| P value |  | P<0.001 |

***S3. Basic characteristic of 65 ears.*** There was no significant difference on PTA and age between the two groups

|  | Total ear (s) | Age (year) | PTA (dB HL) |
| --- | --- | --- | --- |
| SP/AP<34% | 36 | 70.89±5.82 | 46.47±2.60 |
| SP/AP≥34% | 29 | 74.48±7.71 | 45.90±2.85 |
